# Health-Related Quality of Life and Frequency of Physical Activity in Spanish Students Aged 8–14

**DOI:** 10.3390/ijerph18179418

**Published:** 2021-09-06

**Authors:** José I. Calzada-Rodríguez, Ángel M. Denche-Zamorano, Jorge Pérez-Gómez, María Mendoza-Muñoz, Jorge Carlos-Vivas, Sabina Barrios-Fernandez, José Carmelo Adsuar

**Affiliations:** 1 Health, Economy, Motricity and Education (HEME) Research Group, Faculty of Sport Sciences, University of Extremadura, 10003 Cáceres, Spain; jocalzada@alumnos.unex.es (J.I.C.-R.); jorgepg100@unex.es (J.P.-G.); 2 Promoting a Healthy Society Research Group (PHeSO), Faculty of Sport Sciences, University of Extremadura, 10003 Cáceres, Spain; jorgecv@unex.es (J.C.-V.); jadssal@unex.es (J.C.A.); 3 Social Impact and Innovation in Health (InHEALTH) Research Group, Faculty of Sport Sciences, University of Extremadura, 10003 Cáceres, Spain; sabinabarrios@unex.es

**Keywords:** physical activity, HRQoL, Spanish National Health Survey, school population, secondary education

## Abstract

The study of health-related quality of life (HRQoL) in children and adolescents has important implications in terms of policy, education, and health. Data on the time spent in physical activity (PA) and in sedentary activities in this population are worrying. We aim to analyze possible differences in HRQoL and PA levels between sexes and age groups in Spanish students aged between 8 and 14 years, as well as to assess the relationship between HRQoL and the frequency of PA in this population. A total of 3197 participants (1610 boys and 1587 girls) from 8 to 14 years old were recruited. Mquality and Mapping Child Health Utility instrument (Chu9d) were used as HRQoL indicators. A medium positive association between PA and HRQoL concerning the Spanish school population was found. HRQoL was higher among students aged 8 to 12 than 13 to 14. Moreover, when children start secondary education, both sexes seem to lose the quality of life. Similarly, PA decreases among girls over the years, although it seems to increase among boys. Thus, PA levels and HRQoL are directly associated in Spanish schoolchildren aged between 8 and 14 years. However, this HRQoL decreases in children over the years. Practical implications include the need to support education and physical activity programs to improve HRQoL in children and adolescents.

## 1. Introduction

Quality of life (QoL) is a broad multicomponent concept that captures positive and negative elements of well-being, including facets of personal health, education and work, social relationships, economic status and safety, among others [1]. Specifically, Health-Related Quality of Life (HRQoL) focuses on the individuals’ subjective self-perception about their current health status and ability to perform daily activities in different life domains [2,3], considering individuals’ subjective insights of their own well-being, physical, social and mental health, and functioning. HRQoL erects an important indicator of overall health, not only for the general population but for clinical individuals as well, being useful to measure changes after interventions or treatments [4]. Moreover, HRQoL represents the most common approach to assess QoL since it covers a wide range in health status, including subjective perceptions and thoughts, and individuals’ functioning and capacity to develop in different life domains [5,6].

Studying children’s QoL is imperative because, in addition to being a moral and legal imperative, its development will determine later stages of their lives, including adolescence and adulthood [5,7]. Moreover, children’s physical, psychological and social functioning in accordance with their developmental progress, individual differences and socio-cultural context must be considered. It includes their ability to perform activities of daily living; to interact and play with peers and family members; the characteristics of their home and family environment; their cognitive, emotional and behavioral management skills; their energy and vitality; and their perception about their own health, among others [8].

The World Health Organization (WHO) defines overweight and obesity as a chronic disease characterized by abnormal or excessive body fat accumulation that represents a risk for health [9]. Their consequences include physical (increased risk of cardiovascular problems, type 2 diabetes and adulthood obesity), psychological (anxiety, depression, lower self-esteem) and social (social stigma) issues [10]. Childhood obesity and overweight have become a global public health problem since 337 million children or adolescents were living with obesity in 2020 [11]. Spain is one of the countries that presents the highest overweight and obesity incidence in Europe and worldwide [12]. The ALADINO studies carried out in 2015 and 2019 [13,14] found that high levels of inactivity were related to wrong nutrition patterns, too much screen time or socio-economic difficulties. Additionally, children with unhealthy behaviors usually have poor diets and insufficient levels of physical activity (PA) [15]. Tsiros et al. [7] found an inverse association between body mass index (BMI) and HRQoL in children, which may be related to the abovementioned factors. Furthermore, although sedentary lifestyle is growing among children and adolescents, there is limited evidence about its relationship with HRQoL. It may be due to the screen-based media time, which represents a high percentage of free-time use among children and adolescents, replacing outdoor play in some cases [16]. Current examples of sedentary behavior with reduced energy expenditure that usually implies screen-based activities, such as watching TV or computer tasks, are associated with lower fitness. Goldfield et al. [17] stated that screen time is inversely linked with general and psychological HRQoL in young populations with overweight and obesity. Lacy et al. [16] also found that higher screen use involved lower HRQoL in Australian adolescents, while PA represented a HRQoL positive indicator. Therefore, huge screen time should be related to negative health issues, such as obesity, lower HRQoL and poorer physical and psychosocial well-being [18].

Considering the multidimensional nature of HRQoL, different research has been carried out in the field of PA, showing improvements at physical, psychological, cognitive and social levels in healthy children [19], healthy adolescents [20], and healthy adults [21], as well as in others with diverse pathologies [22,23,24,25,26,27,28,29,30]. Many studies have linked PA to psychological and cognitive function improvements in children and adolescents [31,32]. Therefore, we aim to analyze if there exist differences in HRQoL and PA levels between sexes and age groups in Spanish students aged 8 to 14 years as well as to explore the relationship between HRQoL and the frequency of PA practice in this population.

## 2. Materials and Methods

### 2.1. Study Design

A descriptive correlational study was conducted. The Spanish National Health Survey (ENSE 2017) [33] provided data to proceed with the selection of the sample. This survey was developed conducting interviews with children’s parents/guardians. Firstly, the Ministry of Health, Consumer Affairs and Social Welfare sent a letter to randomly selected households for initial contact. In that letter, collaboration was requested providing information about the procedure and consent for an interviewer to visit them. In addition, information about demographic data and health issues was required in two different parts of the interview. Data were collected through a computer-assisted personal interview, which could be replaced by a telephone interview if necessary. The National Statistical Institute supervised data processing.

### 2.2. Participants

To be included in the study, participants needed to meet the following eligibility criteria: (a) children aged between 8 and 14 years old; (b) including all records in the variables needed to calculate the Mapping Child Health Utility instrument (Chu9d) (module E) and Mquality, which are measures of children’s quality of life. Answers of “don’t know” and “not available” were classified as “missing data” for both module E questions and K61. Thus, initially, 6106 participants were interviewed; however, only 3197 participants (1610 boys and 1587 girls) aged 8–14 years, with a mean age of 11.16 (±1.97) years met the eligibility criteria and composed the study final sample.

### 2.3. Procedures

As mentioned above, the Ministry of Health, Consumer Affairs, and Social Welfare, in collaboration with the National Statistics Institute, was responsible for designing the abovementioned national survey, ENSE 2017 [33]. This survey allowed us to know aspects of citizens’ health at a national and regional level, as well as to plan evaluations on health matters including three questionnaires: home, adult, and child. Data obtained from children were collected through interviews with the parents. Thus, the questionnaire for children was applied to analyze their HRQoL based on their self-perception about physical, mental and social health, using the Screening for and Promotion of Health Related Quality of Life in Children and Adolescents—a European Public Health Perspective (Kidscreen-10) instrument adapted from Eurobarometer [34,35,36], with one question less than the original version.

Only two modules of ENSE 2017 [33] were considered in this study:

- **“Module E: Quality of Life”.** This evaluates children’s self-perception about their HRQoL, involving subjective physical, mental and social status. The Kidscreen-10 modified instrument was used, according to its parental version. Question 7 (“Have the parents of the child or adolescent treated him/her fairly?”) was specifically removed from the primary version. The questions included assess the frequency in which the child or adolescent had experienced the following situations over the last week: (1) has felt good and in good shape; (2) has been full of energy; (3) has felt sad; (4) has felt lonely; (5) has had enough time for him/herself; (6) has been able to do desired things in free time; (7) has had fun with friends; (8) has done well at school; and 9) has been able to pay attention. The possible response levels were: (1) “nothing”, (2) “a little”, (3) “moderately”, (4) “very much”, (5) “a lot”. Either the primary version of the instrument and its modified 9-item version have indicated good reliability in European children (Cronbach’s α = 0.82 and 0.75, respectively) [35,36]. Moreover, its Spanish version has proven to be valid and reliable (Cronbach’s α > 0.70) [37].
- **“Module K: Rest and Physical Activity”.** This was used for children’s physical activity frequency assessment. Only question 61, which is related to leisure-time physical activity, was considered. Thus, the primary objective was to quantify physical activity volume to appraise whether children met the WHO recommendations on PA. For that purpose, the short version of the adapted International Physical Activity Questionnaire (IPAQ) was used, which has shown good psychometric properties (Spearman’s *p* = 0.80 and Cronbach’s α > 0.80) [38]. Possible answers for question 61 were: (1)“no exercise” (free-time mainly occupied by sedentary activities such as reading, watching television, going to the cinema…); (2) “occasional physical activity or sport” (walking or cycling, gentle gymnastics, recreational activities that require a slight effort…); (3) “physical activity several times a month” (sports, gymnastics, running, swimming, cycling, team games…); and (4) “sports or physical training several times a week”.

**Figure 1:**
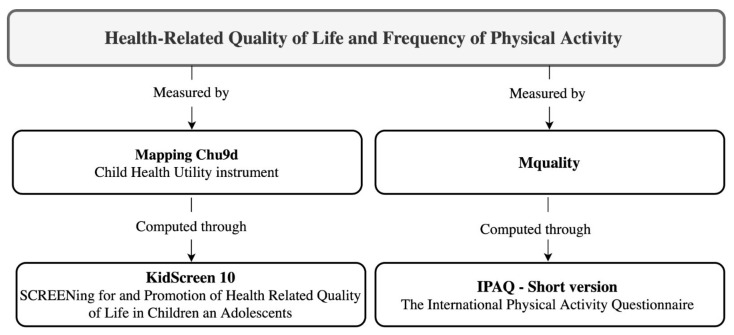
Schematic diagram of main variables and constructs of the study.

Figure 1 shows a schematic diagram to illustrate the variables/constructs of interest.

### 2.4. Health-Related Quality of Life and Physical Activity Outcomes

- **Mapping Child Health Utility instrument (Chu9d).** This instrument evaluates HRQoL by processing the scores obtained from the questions included in “Module E: Quality of Life” (E14_1, E14_2, E14_3, E14_4, E14_8 and E14_9), extracted from the Kidscreen-10 instrument adapted for the Eurobarometer [36]. The variable is calculated according to Chen, Stevens, Rowen and Ratcliffe’s formula [39], appropriate for economic evaluations of health care since it transforms health status into social preferences. Moreover, it has been used in previous research on the same survey and database [40]. Results were collected on a scale of values of 0 to 1, being 0 the minimum and 1, the maximum values of HRQoL.
- **Mquality.** This variable is provided by the ENSE 2017 [33] and directly assesses children’s HRQoL. Calculations were implemented from the “Module E: Quality of Life” questions results, which correspond to the Kidscreen-10 Index modified proxy for the Eurobarometer. Nine questions presented a recall period of one week and five categories of responses as a Likert scale, with question number 10 the general perceived health question. The scores of the abovementioned instrument were transformed into a 0–100 scale that is used in the European Eurobarometer study: higher scores match higher HRQoL.
- **Physical Activity (K61 in ENSE 2017).** This instrument determines the PA done by the child in his/her free time, regarding the frequency of engagement. The final score was accomplished through the analysis of the response level in question 61 of “Block K: Rest and Physical Activity”. Possible values were: (1) “the child does not exercise”, (2) “the child does some occasional physical activity or sport”, (3) “the child does physical activity several times a month”, or (4) “the child does sports or physical training several times a week”.

### 2.5. Statistical Analysis

Statistical procedures and analysis were conducted using the Statistical Package for the Social Sciences (SPSS, Version 25, IBM SPSS, Armonk, NY, USA) software.

The factor variable for weighting the sample was not entered due to missing data. Data are presented as mean and standard deviation (SD), and median and interquartile range (IR). The Kolmogorov–Smirnov test was applied to check data normality in all variables (age, PA, Mquality and Mapping). Any variable followed a parametric distribution.

The sample was subdivided by sex, and age (8–12 years and 13–14 years). Then, The Mann–Whitney U test was conducted for between-sex comparisons, as well as for contrasting age group within sex. Additionally, Spearman’s correlation was run, including Mapping Chu9d, Mquality and PA for assessing the level of association between variables. Correlation coefficients were interpreted following the threshold proposed by Mondragón [41]: 0.00, no correlation; 0.01 to 0.10, weak; 0.11 to 0.50, medium; 0.51 to 0.75, considerable; 0.76 to 0.90, very strong; and 0.91 to 1.00, perfect. Alpha level was set at *p* < 0.05.

Additionally, the Mann–Whitney U-test was applied to verify possible differences at baseline. Then, post hoc analyses were performed for pairwise comparisons after multiple-comparison checking through the Kruskal–Wallis test. Thus, Bonferroni’s correction was required to provide greater accuracy to the analysis, considering *p* < 0.01 as statistically significant.

Finally, the Mann–Whitney U-test was also implemented for assessing differences in HRQoL between those students who do not participate in physical activity and engage occasionally/several times a month (values 1, 2 and 3 in PA) and those who engage in physical activity several times a week (value 4 in PA).

## 3. Results

Table 1 shows the descriptive statistics for the total sample (*n* = 3197) stratified by sex and age ranges. Significant differences were observed in Mquality scores between total sample age subgroups (*p* < 0.001) and between age subgroups in girls (*p* < 0.001). No significant differences were found between sexes. Similarly, meaningful differences were also found in Mapping Chu9d between age subgroups considering the total sample (*p* < 0.001) and only girls (*p* < 0.001). Moreover, significant differences between age ranges were obtained in boys (*p* = 0.034) and between sexes (*p* = 0.039).

HRQoL decreases in total sample and girls as they grow up, being more pronounced among girls. Regarding the 8–12 years subgroup, girls showed higher HRQoL than boys, while it became similar at 13–14 years.

PA outcomes indicate that subjects perform PA several times a month. Significant differences were found between sexes (*p* < 0.05), being the frequency of PA practices lightly lower in girls as they grow up.

Table 2 displays the comparison between subgroups of performed physical activity in HRQoL from Mquality and Mapping Chu9d. Significant differences between subgroups were found in HRQoL. Both Mquality and Mapping Chu9d increased as higher levels of physical activity were achieved, following the established values (from 1 to 4), except for the combination between levels 2 and 3. The Kruskal–Wallis test was implemented for both studied variables to evaluate significant differences in HRQoL levels, applying the previously mentioned response levels of PA as factors, showing significant differences (*p* < 0.001). Then, significant differences are shown between all the possible subgroup combinations in HRQoL (both Mquality and Mapping Chu9d), except in the 2 vs. 3 subgroups comparison (*p* = 0.04 and *p* = 0.956, respectively).

Additionally, HRQoL was determined by comparing students who did not participate in physical activity and engage occasionally/several times a month (values 1, 2 and 3 in PA) with those who engage in physical activity several times a week (value 4 in PA) (Table 3). Significant differences were found between groups for both variables (*p* < 0.001), confirming that HRQoL differs between students who engage in physical activity several times a week and the others.

Table 4 shows the association between HRQoL and PA for the total sample and stratified by sex and age. Spearman’s correlation coefficient was calculated both for the whole sample and independently for sexes and age subgroups within sex. A direct positive correlation between HRQoL and PA was obtained independently of the indicator used (Mquality: r = 0.124; *p* < 0.001; and Mapping Chu9d r = 0.122; *p* < 0.001) for the total sample. Significant correlations were also observed for boys and girls, separately.

Significant medium correlations were observed between PA with Mquality (r = 0.124; *p* < 0.001) and Mapping Chu9d (r = 0.122; *p* < 0.001) in the total sample; and for boys (r = 0.142; *p* < 0.001) and girls (r = 0.123, *p* < 0.001) separately. Considering age ranges and sex, significant medium positive correlations were observed between PA and HRQoL in 8–12 years (r = 0.119–0.130; *p* < 0.001) and 13–14 years (r = 0.173–0.201; *p* < 0.001) boys. Similarly, 13–14 years girls outcomes show a medium association between PA and HRQoL (r = 0.153–0.174; *p* = 0.001 to < 0.001); however, this relationship was only weak in 8–12 years girls (r = 0.089–0.100; *p* = 0.003 to 0.001).

## 4. Discussion

This study provides information regarding the link between HRQoL and PA among Spanish children and adolescents through the ENSE 2017 that should encourage stakeholders to develop actions focused on PA and health promotion, in line with previous reports [40].

On the one hand, regarding sexes, we found that boys engage in PA more frequently when compared to girls. Additionally, HRQoL seems to decrease as children grow up, especially in girls. These results are reinforced according to previous studies related to this topic. One cross-sectional study involving a large sample of European teenagers showed that only a small minority met the WHO recommendations, finding significant differences among sexes, with being boys more engaged in physical and sporting activities than girls [42]. Furthermore, although our results only reported slight differences between sexes, literature determines distinctions based on gender in PA that influence young girls [43,44] primarily associated with lower support from their family and closest social environment. Low fitness, higher body fat percentage and low perceived competence seem to influence girls when engaging in PA [45].

On the other hand, according to age groups, differences in HRQoL were found, especially when moving from primary to secondary education. Several studies on children have documented a trend of decreasing HRQoL with age [46,47,48,49,50,51,52], so results are in line with previous scientific literature. Considering the link between HRQoL and PA, organized sports provide an opportunity for enhancing young people’s health through PA [53]. However, children seem to give up sports-related activities when starting high school [54,55]. This PA reduction could be influenced by puberty since it represents complex phase changes. Rutten, Boen and Seghers [56] found alterations when children progress to secondary education, based on a decline in PA and increased time in sedentary activities (technology and homework). Other studies have found that increased demotivation towards physical sports among high-school students, in contrast to higher rates of intrinsic and external motivation, was demonstrated in primary-education children [57,58]. Hence, the reasons for PA and HRQoL decreasing when starting secondary education could be related to puberty changes, lower motivation to exercise and do sports, and increased time in sedentary activities.

Overall, HRQoL seems to improve as students are included in higher PA levels (Table 2 and Table 3). This can be explained due to the similarities between levels. Likewise, statistically significant differences in HRQoL are reported between students who do not engage in any kind of exercise/physical activity or do it irregularly and those who do it several times a week (Table 4). Several studies found similar outcomes, showing a dose-response when linking physical activity levels and HRQoL in children and adolescents. Thus, greater HRQoL is achieved when levels of PA get higher (or sedentary behaviors are reduced) [16,59,60,61,62,63]. These outcomes also make sense according to the established PA guidelines for health for young population, which show health is linked with being physically active virtually daily [64].

PA implies improved HRQoL and great benefits for children and adolescents. PA implemented with a low, moderate and/or vigorous intensity is essential to encourage health promotion. High-intensity PA is related to greater physical fitness, bone health, motor skills and cardiometabolic biomarkers, among other indicators, in children and adolescents [19]. Moreover, cardiorespiratory fitness and musculoskeletal fitness are connected to HRQoL [65]. Since PA influences children’s physical characteristics/properties and has positive effects on well-being, their HRQoL increases too.

In addition, PA benefits not only young people’s fitness but also their cognition and mental processes [66]. In contrast, sedentary behaviors impair not only fitness and body composition but also lead to reduced self-esteem [67] and depression [68]. Increased screen-based activities promote sedentariness and are related to poorer mental health [69], which is directly linked to HRQoL [70]. Biddle and Asare [31] showed that PA can improve children’s mental health, so intervention programs based on exercise may be beneficial for children’s and adolescents’ mental health. Hence, sports include specific characteristics of cooperation and socialization, affecting youngsters’ self–esteem and social interaction skills [71].

Based on the abovementioned, there is no doubt that PA enhances children’s HRQoL. Thus, it becomes necessary to implement practical initiatives for health promotion through PA. Therefore, it may be of interest to advise the practice of exercise and sports several times per week, as physical inactivity represents a key factor for chronic diseases, while being physically active helps to prevent and delay their effects [72]. As schools represent the main place for social and physical development of children and adolescents, Physical Education (PE) becomes a useful tool to motivate young people to engage in PA. Likewise, physical exercise programs can encourage young people to develop and enjoy physically active behaviors that could be maintained through life and be transferred to many daily-life contexts, raising at the same time the HRQoL [73]. This research may help PA and health-promotion professionals to have a greater knowledge about the importance of preserving young people’s subjective health status and well-being. This information is valid for children and their families, but also for stakeholders involved in physically active practices.

The main limitation of this study is that cause–effect association cannot be obtained. However, observational studies can help in suggesting hypotheses about those causal relationships that shall be tested in further studies [74]. In our case, experiments regarding PA and HRQoL should be carried out, focusing on how variables could be influenced (such as individuals’ characteristics or socioeconomic status), how regular physical activity could improve it (exploring needed mechanisms) and to what extent the start of secondary education negatively interacts with physical activity levels among the studied population. Likewise, it would be interesting that future studies explore the association between HRQoL and the parameters that reflect the motor capacity of children.

## 5. Conclusions

HRQoL decreases in children over the years, and this decrease is greater in girls. Although the differences found are slight, it can be stated that engaging in exercise/physical activity several times per week is related to a higher HRQoL. It seems that HRQoL may decrease when children start secondary education. Moreover, there exists a direct association between frequency of engagement in PA and HRQoL in Spanish schoolchildren aged between 8 and 14 years.

## Figures and Tables

**Table 1:** Descriptive statistics for age, HRQoL and physical activity for total sample and by sex and age.

Variables	Total			*p* *	Boys			*p* *	Girls			*p* *	*p* **
Age	8–12 (*N* = 2205)	13–14 (*N* = 992)	8–14 (*N* = 3197)		8–12 (*N* = 1119)	13–14 (*N* = 491)	8–14 (*N* = 1610)		8–12 (*N* = 1086)	13–14 (*N* = 501)	8–14 (*N* = 1587)		
Median (IR)	10.00 (2)	13.00 (1)	11.00 (4)	n/a	10.00 (2)	13.00 (1)	11.00 (4)	n/a	10.00 (2)	14.00 (1)	11.00 (3)	n/a	0.448
Mean (SD)	10.11 (1.41)	13.50 (0.50)	11.16 (1.97)		10.10 (1.40)	13.49 (0.50)	11.14 (1.97)		10.12 (1.42)	13.50 (0.50)	11.19 (1.98)		
**PA**													
Median (IR)	3.00 (2)	3.00 (2)	3.00 (2)	0.526	3.00 (2)	3.00 (2)	3.00 (2)	0.041	3.00 (2)	3.00 (2)	3.00 (2)	0.008	<0.001
Mean (SD)	2.90 (1.03)	2.85 (1.09)	2.88 (1.05)		3.04 (0.98)	3.12 (1.00)	3.06 (0.99)		2.75 (1.06)	2.59 (1.12)	2.70 (1.08)		
**Mquality**													
Median (IR)	90.00 (12.50)	87.50 (15.00)	90.00 (12.50)	<0.001	90.00 (12.50)	87.50 (15.00)	90.00 (12.50)	0.085	90.00 (12.50)	87.50 (15.00)	90.00 (12.50)	<0.001	0.266
Mean (SD)	87.98 (10.30)	86.23 (11.20)	87.43 (10.62)		87.57 (10.59)	86.61 (10.70)	87.27 (10.63)		88.40 (9.97)	85.86 (11.67)	87.60 (10.60)		
**Mapping Chu9d**													
Median (IR)	0.99 (0.10)	0.97 (0.12)	0.97 (0.11)	<0.001	0.97 (0.11)	0.97 (0.11)	0.97 (0.11)	0.034	0.99 (0.11)	0.97 (0.12)	0.98 (0.10)	<0.001	0.039
Mean (SD)	0.97 (0.08)	0.96 (0.09)	0.97 (0.08)		0.97 (0.08)	0.96 (0.09)	0.97 (0.08)		0.98 (0.08)	0.96 (0.09)	0.97 (0.08)		

IR: interquartile range; SD: Standard deviation; Mapping Chu9d: Mapping Child Health Utility instrument; PA: Physical activity; *p* *: Between-age multiple comparison *p*-value for the total sample and for boys and girls, respectively; *p* **: Between-sex comparison *p*-value.

**Table 2:** Relationship between HRQoL and PA practice frequency.

PA		Mquality	PA	Medians Dif	Means Dif	p *	p **	Mapping Chu9d	PA	Medians Dif	Means Dif	p *	p **
1	Median (IR)	87.50 (15)	2	−2.50	−3.16	<0.001	<0.001	0.967 (0.117)	2	−0.006	−0.019	<0.001	<0.001
	Mean (SD)	84.23 (12.46)	3	0.00	−2.68		0.002	0.944 (0.967)	3	−0.006	−0.025		<0.001
			4	−2.50	−4.93		<0.001		4	−0.027	−0.037		<0.001
2	Median (IR)	90 (17.5)	1	2.50	3.16	<0.001	<0.001	0.973 (0.111)	1	0.006	0.019	<0.001	<0.001
	Mean (SD)	87.39 (11.17)	3	2.50	0.48		0.04	0.963 (0.09)	3	0.000	−0.005		0.956
			4	0.00	−1.77		0.006		4	−0.021	−0.018		<0.001
3	Median (IR)	87.50 (15)	1	0.00	2.68	<0.001	0.002	0.973 (0.107)	1	0.006	0.025	<0.001	<0.001
	Mean (SD)	86.91 (9.74)	2	−2.50	−0.48		0.04	0.968 (0.077)	2	0.000	0.005		0.956
			4	−2.50	−2.25		<0.001		4	−0.021	−0.013		<0.001
4	Median (IR)	90 (12.5)	1	2.50	4.93	<0.001	<0.001	0.995 (0.108)	1	0.027	0.037	<0.001	<0.001
	Mean (SD)	89.16 (9.88)	2	0.00	1.77		0.006	0.981 (0.073)	2	0.021	0.018		<0.001
			3	2.50	2.25		<0.001		3	0.021	0.013		<0.001

IR: interquartile range; SD: Standard deviation; Mapping Chu9d: Mapping Child Health Utility instrument; PA: Physical activity. * Global Kruskal–Wallis for *p* < 0.001 was obtained through using either Mquality or Mapping Chu9d (HRQoL scores) as an answer and PA levels from 1 to 4 (frequency of physical activity performed by the child) as a factor. ** Post hoc analysis applying Bonferroni’s correction factor in the Mann–Whitney U test having *p* = 0.01. Mquality: variable that assesses HRQoL levels by a score from 0 to 100. Mapping Chu9d: variable that evaluates HRQoL on a 0–1 scale. PA: frequency of physical activity performed by the child whose value is collected on a scale of 1 to 4 (established response levels for K61). Mean dif: the difference between the average values of Mquality or Mapping Chu9d variables for each PA level (from 1 to 4). Medians dif: the difference between the medians of Mquality or Mapping Chu9d for each PA level (from 1 to 4).

**Table 3:** Analysis of HRQoL values for the Mquality and Mapping Chu9d variables by comparing students who do little or no physical activity and those who do physical activity several times a week.

Variables	Not Participate in Physical Activity or Do Occasionally/Several Times a Month (*N* = 2046)	Participate in Physical Activity Several Times a Week (*N* = 1151)	*p*
Mquality			<0.001
Median (IR)	87.50 (15)	90 (12.5)	
Mean (SD)	86.47 (10.89)	89.16 (9.89)	
Mapping Chu9d			<0.001
Median (IR)	0.97 (0.111)	0.99 (0.108)	
Mean (SD)	0.96 (0.086)	0.98 (0.073)	

IR: interquartile range; SD: Standard deviation; Mapping Chu9d: Mapping Child Health Utility instrument. Non-parametric Mann–Whitney U test for *p* < 0.001. Mquality: variable that assess HRQoL levels by a score from 0 to 100. Mapping Chu9d: variable that evaluates HRQoL on a 0–1 scale. Do not participate in physical activity or do it occasionally/several times a month: values 1–3 in PA. Do participate in physical activity several times a week: value 4 in PA.

**Table 4:** Association between HRQoL (Mquality and Mapping Chu9d) and PA.

Mquality	PA *rho*	*p*	Mapping Chu9d	PA *rho*	*p*
Total sample	0.124	<0.001	Total sample	0.122	<0.001
Boys (total)	0.142	<0.001	Boys (total)	0.142	<0.001
8–12 years	0.130	<0.001	8–12	0.119	<0.001
13–14 years	0.173	<0.001	13–14	0.201	<0.001
Girls (total)	0.123	<0.001	Girls (total)	0.123	<0.001
8–12 years	0.089	0.003	8–12	0.100	0.001
13–14 years	0.174	<0.001	13–14	0.153	0.001

Mapping Chu9d: Mapping Child Health Utility instrument as the variable that evaluates HRQoL on a 0–1 scale; PA: Physical activity, as frequency of physical activity performed by the child whose value is collected on a scale of 1 to 4 (established response levels for K61). Mquality: variable that assesses HRQoL levels by a score from 0 to 100.

## Data Availability

The datasets used during the current study are available from the corresponding author on reasonable request.
